# Hepatitis C virus infection and risk of liver-related and non-liver-related deaths: a population-based cohort study in Naples, southern Italy

**DOI:** 10.1186/s12879-021-06336-9

**Published:** 2021-07-08

**Authors:** Pierluca Piselli, Diego Serraino, Mario Fusco, Enrico Girardi, Angelo Pirozzi, Federica Toffolutti, Claudia Cimaglia, Martina Taborelli, on behalf of the Collaborating Study Group, Mariarosaria Capobianchi, Fabrizio Carletti, Anna R. Garbuglia, Giuseppe Ippolito, Paola Scognamiglio, Pietro Di Cicco, Letizia Gigli, Silvana Russospena, Raffaele Palombino, Chiara Panato, Veronica Mattioli, Luigino Dal Maso

**Affiliations:** 1 Department of Epidemiology and Pre-Clinical Research, National Institute for Infectious Diseases “L. Spallanzani”, Rome, Italy; 2 Cancer Epidemiology Unit, Centro di Riferimento Oncologico di Aviano (CRO) IRCCS, via Franco Gallini 2, 33081 Aviano, PN Italy; 3 Registro Tumori, ASL Napoli-3 Sud, Brusciano, Naples, Italy

**Keywords:** Hepatitis C virus, Liver disease, Mortality, Cohort study, Southern Italy

## Abstract

**Background:**

Hepatitis C virus (HCV) infection represents a global health issue with severe implications on morbidity and mortality. This study aimed to evaluate the impact of HCV infection on all-cause, liver-related, and non-liver-related mortality in a population living in an area with a high prevalence of HCV infection before the advent of Direct-Acting Antiviral (DAA) therapies, and to identify factors associated with cause-specific mortality among HCV-infected individuals.

**Methods:**

We conducted a cohort study on 4492 individuals enrolled between 2003 and 2006 in a population-based seroprevalence survey on viral hepatitis infections in the province of Naples, southern Italy. Study participants provided serum for antibodies to HCV (anti-HCV) and HCV RNA testing. Information on vital status to December 2017 and cause of death were retrieved through record-linkage with the mortality database. Hazard ratios (HRs) for cause-specific mortality and 95% confidence intervals (CIs) were estimated using Fine-Grey regression models.

**Results:**

Out of 626 deceased people, 20 (3.2%) died from non-natural causes, 56 (8.9%) from liver-related conditions, 550 (87.9%) from non-liver-related causes. Anti-HCV positive people were at higher risk of death from all causes (HR = 1.38, 95% CI: 1.12–1.70) and liver-related causes (HR = 5.90, 95% CI: 3.00–11.59) than anti-HCV negative ones. Individuals with chronic HCV infection reported an elevated risk of death due to liver-related conditions (HR = 6.61, 95% CI: 3.29–13.27) and to any cause (HR = 1.51, 95% CI: 1.18–1.94). The death risk of anti-HCV seropositive people with negative HCV RNA was similar to that of anti-HCV seronegative ones. Among anti-HCV positive people, liver-related mortality was associated with a high FIB-4 index score (HR = 39.96, 95% CI: 4.73–337.54).

**Conclusions:**

These findings show the detrimental impact of HCV infection on all-cause mortality and, particularly, liver-related mortality. This effect emerged among individuals with chronic infection while those with cleared infection had the same risk of uninfected ones. These results underline the need to identify through screening all people with chronic HCV infection notably in areas with a high prevalence of HCV infection, and promptly provide them with DAAs treatment to achieve progressive HCV elimination and reduce HCV-related mortality.

**Supplementary Information:**

The online version contains supplementary material available at 10.1186/s12879-021-06336-9.

## Background

In 2015, the global prevalence of hepatitis C virus (HCV) infection was estimated to be 1%, corresponding to 71.1 million people living with HCV [1]. In Italy, about 850-thousand people are supposed to be chronically infected with HCV (of whom only 40% are aware of being infected) [2], and a strong geographic gradient with elevated prevalence in the southern part of the Country has been consistently reported [3, 4].

Among people infected with HCV*,* between 55 and 85% will progress to chronic HCV infection, a condition that puts them at risk of liver cirrhosis, liver failure, and hepatocellular carcinoma (HCC). Since HCV infection is often asymptomatic, most cases remain undiagnosed until the very late stages of liver diseases. A considerable number of data suggest that the use of Direct-Acting Antiviral (DAAs) therapies -with up to 95% rates of sustained virological response in patients, including those previously excluded from interferon-based regimens- could reverse the rising trends in HCV incidence and HCV-related morbidity and mortality [5–7]. To this regard, it is worth mentioning that the World Health Organization (WHO) targets for 2015–2030 include the reduction of new HCV infections by 80%, HCV deaths by 65%, increasing HCV diagnoses to 90% and the number of eligible persons who receive HCV treatment up to 80% [8].

In Italy, approximately 180,000 patients were administered second and third generation DAA between December 2014 and December 2018 with a 95–96% estimated overall response [9]. Up to March 2017, the National Healthcare provided free access to therapy for patients affected by HCV-related cirrhosis, advanced fibrosis, and other severe comorbidities. Thereafter, all HCV RNA positive patients were permitted access to DAAs [10]. Currently, in line with both European and American guidelines, in Italy provisions and reimbursement for DAAs therapies are universally available to all patients with documented chronic HCV infection, providing they have no contraindications to therapy, such as limited life expectancy due to severe comorbidities [11].

The burden of HCV-infection on all-cause and on liver-related deaths has been recently described by the Global Burden of Disease Study [9]. In 2017, approximately 235,000 deaths worldwide were attributable to HCV-related liver cancer, with a 30.4% increase between 2007 and 2017. With regard to Italy, the International Agency for Research on Cancer (IARC) estimated that about 60% of liver cancer cases were infected with HCV [12]. Moreover, an Italian case-control study reported that 61% of HCC cases were attributable to HCV infection, 13% to hepatitis B virus (HBV) infection, and 18% to heavy alcohol consumption [13].

Incidence and mortality for liver cancer and the prevalence of HCV and of HBV infection are particularly elevated in southern Italy. A population-based investigation conducted by our study group between 2003 and 2006 showed that 23% of people aged 65 or more were HCV-positive in the province of Naples, an area with high incidence rates of liver cancer [14]. In the present investigation, we took advantage of that population-based study to assess the role of HCV infection on the risk of all-cause, liver-related, and non-liver-related deaths among the cohort members living in an area with a high prevalence of HCV infection before the advent of DAAs. Furthermore, this study aimed to evaluate factors associated with cause-specific mortality among HCV-infected individuals.

## Methods

This ongoing population-based cohort study started in 2003 in the province of Naples (650,000 inhabitants), southern Italy. Study design, methodology, and preliminary results have been already described in detail elsewhere [14, 15]. Briefly, the study enrolled 4496 persons ≥20 years randomly selected from the resident population between October 2003 and March 2006. Study participants were interviewed at enrolment, and they were tested for HCV, HBV infections, and hepatic metabolic activity. The major characteristics of the original cohort are presented in Supplementary Table 1. Out of the 4496 cohort members, 4160 were negative for antibodies against HCV (anti-HCV^─^) and 336 were positive (anti-HCV^+^). These 336 persons were stratified in two groups according to HCV infection status: 90 had a cleared HCV infection (anti-HCV^+^ and HCV RNA^─^); whereas 246 were chronically infected (anti-HCV^+^ and HCV RNA^+^).

**Fig. 1:**
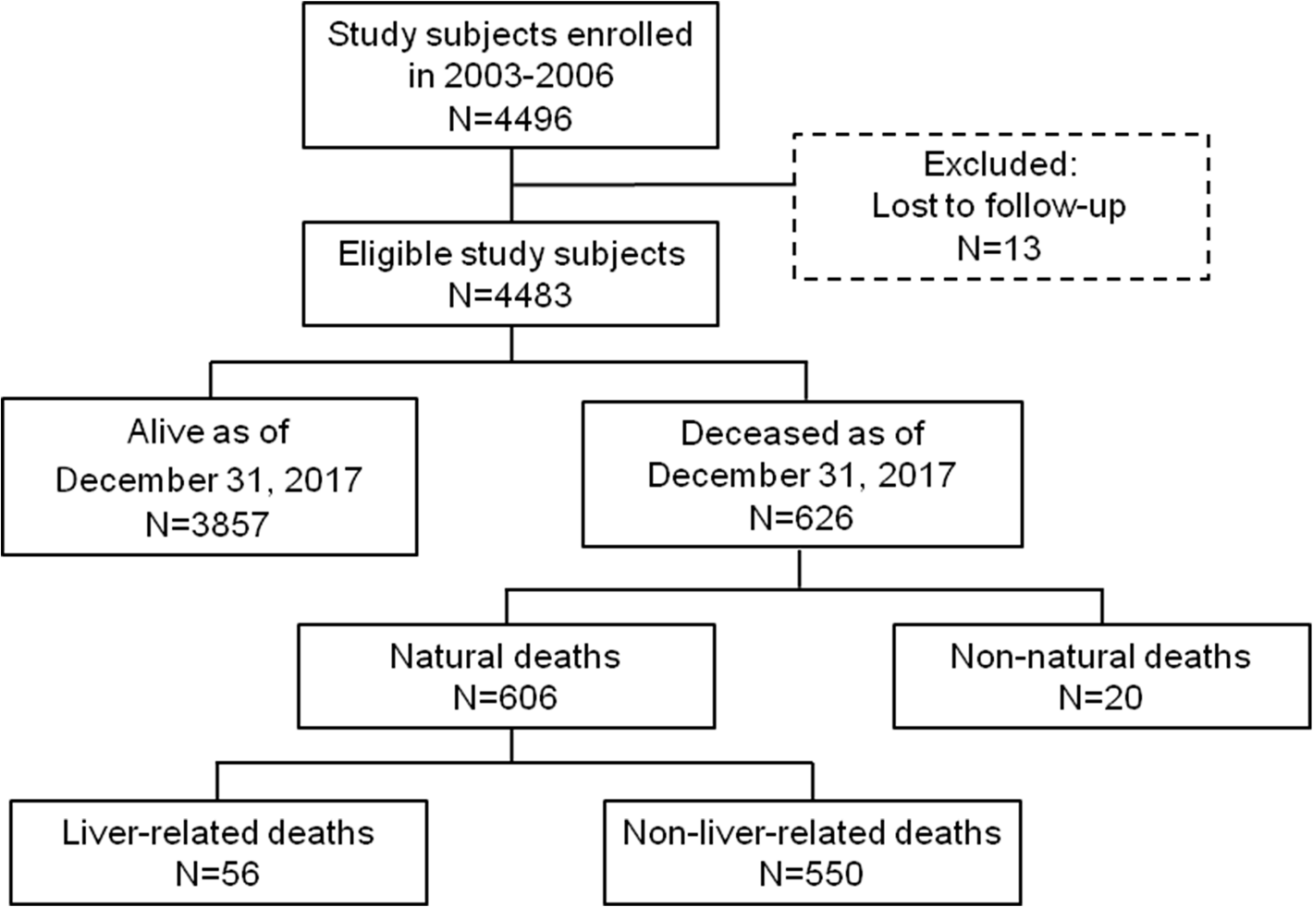
Flow chart of cohort participants enrolled in 2003–2006 and followed-up to December 31, 2017

For the purposes of this analysis, 13 study participants were excluded because they lacked follow-up information, thus leaving 4483 eligible individuals for the present analysis (Fig. 1). Variables collected at enrolment and diagnostic testing for HCV infection have been illustrated in a previous paper [14]. The vital status and, eventually, the cause of death of study participants were ascertained up to December 31, 2017, through a blind record linkage procedures between the cohort database and the regional mortality database of people residing in the study area [15, 16]. The underlying cause of death was classified according to the International Classification of Diseases and Related Health Problems, 10th revision (ICD-10). Analyses on all-cause mortality included all causes of death excluding non-natural ones (i.e., ICD-10 codes: S00-T98). Death causes ascribed to malignant neoplasm of liver (ICD-10 code: C22), viral hepatitis (ICD-10 codes: B15–B19), alcoholic liver disease (ICD-10 code: K70), and non-alcoholic liver diseases (ICD-10 codes: K71–K77) were classified as liver-related, while the remaining natural causes as non-liver related (after classifying causes coded S00-T98 as accidental non-natural causes). Among non-liver related death causes, we separately analyzed those due to circulatory system diseases (ICD-10 codes: I00-I99).

According to data from the United States population showing that diabetes and chronic renal disease were associated with an elevated risk of death in persons with chronic HCV infection [17], we identified individuals with hyperglycaemia (i.e., those with a fasting plasma glucose ≥126 mg/dl) and individuals with a reduced renal function (i.e., those with the glomerular filtration rate, eGFR < 60 ml/min/1.73m^2^). For this latter group, the eGFR was calculated using the Chronic Kidney Disease-Epidemiology Collaboration 2009 equation based on age, sex, race, and serum creatinine [18].

To assess the presence of liver fibrosis, we used the FIB-4 index [13]. To compare our results with those present in literature we divided the FIB-4 scores into three groups [19, 20]: FIB-4 < 1.45: people without liver fibrosis; FIB-4 between 1.45 and 3.25: mild to moderate fibrosis; and FIB-4 > 3.25: those with severe fibrosis.

The demographic and clinical characteristics of the cohort members were presented as frequencies and were compared through the chi-squared test between groups according to HCV infection status.

Person-years (PYs) at risk of death were computed as the time elapsed from HCV testing date to the date of death, or December 31, 2017, whichever occurred first.

To determine absolute risk estimates of mortality according to HCV infection status, the cause-specific cumulative incidence function for all-cause, liver-related, and non-liver-related deaths was estimated. Each analysis was performed treating all other causes of death as a competing risk. Cumulative mortality differences between groups were assessed by means of Gray’s test [21, 22]. To evaluate factors associated with cause-specific mortality, the hazard ratios (HRs) of death and the corresponding 95% confidence intervals (CIs) were estimated using the Fine and Grey’s regression models [23], adjusted for variables that were statistically significant at univariate analysis such as sex, age at enrolment (per 10-year of increase), years of education (< 9, ≥9), HBV surface antigen status (HBsAg^+^, HBsAg^─^), presence of hyperglycaemia and reduced kidney function (none, hyperglycaemia only, reduced kidney function only, both), FIB-4 index score (< 1.45, 1.45–3.25, > 3.25).

The attributable risk (AR) percent was calculated from the full-adjusted HR obtained from the Fine and Grey’s regression model using the formula AR% = [(HR-1)/HR]× 100 [24].

All tests were two-sided, and a *p*-value < 0.05 was considered statistically significant. All statistical analyses were performed using SAS (SAS Institute, Cary, NC, USA, version 9.4).

## Results

The 4483 participants were followed-up for a total of 52,176.6 PYs, with a median follow-up time of 12.7 years (interquartile range, IQR: 11.9–13.6 years). Overall, 626 died during the study period, 20 (3.2%) of them due to non-natural causes, 56 (8.9%) due to liver-related conditions, and 550 (87.9) due to other causes (Fig. 1).

Table 1 shows the demographic and clinical characteristics of the cohort members according to HCV status. Participants who were anti-HCV positive, and the subgroup of those with chronic infection were more likely to be older, to be less educated, to have a higher FIB-4 score, and to be affected by hyperglycaemia and reduced kidney function than those who were anti-HCV negative.

**Table 1:** Distribution of 4483 study participants according to selected variables and markers of HCV

Characteristics	Overall	Anti-HCV^─^	Anti-HCV^+^		Anti-HCV^+^ and HCV RNA^+^	
	(*n* = 4483)	(*n* = 4147)	(*n* = 336)		(*n* = 246)	
	N (%)	N (%)	N (%)	*p-value* vs Anti-HCV^**─**^	N (%)	*p-value* vs Anti-HCV^**─**^
Sex						
Female	2478 (55.3)	2304 (55.6)	174 (51.8)		128 (52.0)	
Male	2005 (44.7)	1843 (44.4)	162 (48.2)	0.18	118 (48.0)	0.28
Age at enrollment (years)						
< 50	2604 (58.1)	2565 (61.9)	39 (11.6)		26 (10.6)	
50–59	736 (16.4)	680 (16.4)	56 (16.7)		43 (17.5)	
60–69	552 (12.3)	455 (11.0)	97 (28.9)		73 (29.7)	
≥ 70	591 (13.2)	447 (10.8)	144 (42.9)	< 0.01	104 (42.3)	< 0.01
Education (years)^a^						
< 9	2693 (62.0)	2425 (60.2)	268 (84.8)		198 (86.1)	
≥ 9	1654 (38.0)	1606 (39.8)	48 (15.2)	< 0.01	32 (13.9)	< 0.01
HBV						
HBsAg^**─**^	4383 (97.8)	4052 (97.7)	331 (98.5)		241 (98.0)	
HBsAg^+^	100 (2.2)	95 (2.3)	5 (1.5)	0.34	5 (2.0)	0.79
Fasting plasma glucose (mg/dl)^a^						
< 126	4148 (93.1)	3857 (93.6)	291 (86.9)		207 (84.5)	
≥ 126	309 (6.9)	265 (6.4)	44 (13.1)	< 0.01	38 (15.5)	< 0.01
eGFR (ml/min/1.73m^2^)^a^						
< 60	203 (4.6)	165 (4.0)	38 (11.3)		26 (10.6)	
≥ 60	4254 (95.4)	3957 (96.0)	297 (88.7)	< 0.01	219 (89.4)	< 0.01
FIB-4 index score^a^						
< 1.45	3540 (79.6)	3437 (83.6)	103 (30.7)		62 (25.2)	
1.45–3.25	777 (17.5)	622 (15.1)	155 (46.3)		113 (45.9)	
> 3.25	131 (2.9)	54 (1.3)	77 (23.0)	< 0.01	71 (29.9)	< 0.01
Follow-up (years)						
Median (IQR)	12.7 (11.9–13.6)	12.7 (11.9–13.6)	12.1 (7.0–13.2)		11.7 (6.2–13.2)	
Total person-years	52,176.6	48,786.8	3389.8		2375.2	

*Abbreviations*: *eGFR* Estimated Glomerular Filtration Rate, *HCV* hepatitis C virus, *IQR* Interquartile range

^a^The sum does not add up to the total because of missing values

**Fig. 2:**
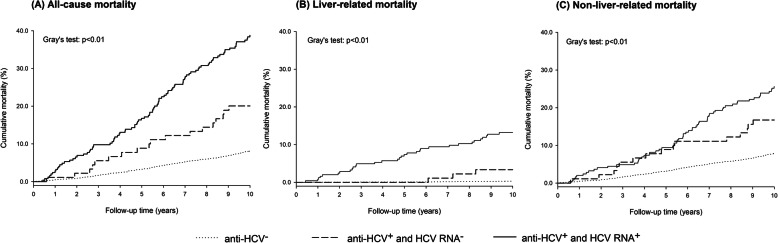
Cumulative mortality for all^a^ (**A**), liver-related (**B**), and non-liver-related^a^ (**C**) causes by HCV markers. Note: ^a^Non-natural causes of death excluded. Abbreviations: HCV, hepatitis C Virus

People with chronic HCV infection showed the highest all-cause cumulative mortality, with nearly a 40% probability of dying after 10 years, as compared to 20% of those with cleared HCV infection and 8% of those who were anti-HCV negative (p for Gray’s test < 0.01) (Fig. 2). These differences were particularly evident for liver-related mortality: the 10-year liver-related mortality for people with chronic HCV infection was 13.2% against 3.3% of those with cleared HCV infection, and 0.3% of those who were anti-HCV negative (p for Gray’s test < 0.01).

Table 2 shows the HRs of death for all-cause, liver-related, and non-liver-related causes, according to HCV status. Compared with HCV-negative participants, those anti-HCV positive were at elevated risk of all-cause deaths (HR = 1.38, 95% CI: 1.12–1.70). In the confirmatory analysis with HCV RNA, cohort members with chronic HCV infection showed a 1.5-fold higher risk of death (95% CI: 1.18–1.94), whereas no excess of risk emerged among those with cleared HCV infection. These effects were magnified for liver-related mortality, where individuals with chronic infection had a 6.6-fold higher risk of death than HCV-negative ones (95% CI: 3.29–13.27). Among anti-HCV positive individuals, 27.5% of all-cause deaths were attributed to HCV infection, an AR percentage that ranged from 8.3% among cohort members with cleared HCV infection to 33.8% among those chronically infected (Table 2). HCV infection had a predominant role in liver-related deaths, with 84.9% of such deaths attributable to HCV among people with chronic infection.

**Table 2:** HRs of death for all, liver-related and non-liver-related causes according to HCV status

HCV status	All-cause deaths^**a**^ (***n*** = 606)			Liver-related deaths (***n*** = 56)			Non-liver-related deaths^**a**^ (***n*** = 550)		
	N (%)	HR (95% CI)^**b**^	AR (95% CI)^**c**^	N (%)	HR (95% CI)^**b**^	AR (95% CI)^**c**^	N (%)	HR (95% CI)^**b**^	AR (95% CI)^**c**^
Anti-HCV^-^	452 (10.9)	1^d^		41 (12.2)	1^d^		437 (10.5)	1^d^	
Anti-HCV^+^	154 (45.8)	1.38 (1.12 – 1.70)	27.5 (10.7 – 41.2)	3 (3.3)	5.90 (3.00 – 11.59)	83.1 (66.7 – 91.4)	113 (33.6)	1.08 (0.84 – 1.37)	7.4 (-19.0 – 27.0)
HCV RNA^-^	31 (34.4)	1.09 (0.77 – 1.54)	8.3 (-29.9 – 35.1)	38 (15.5)	3.05 (0.84 – 11.08)	67.2 (-19.0 – 91.0)	28 (31.1)	1.02 (0.72 – 1.46)	2.0 (-38.9 – 31.5)
HCV RNA^+^	123 (50.0)	1.51 (1.18 – 1.94)	33.8 (15.3 – 48.5)	41 (12.2)	6.61 (3.29 – 13.27)	84.9 (69.6 – 92.5)	85 (34.6)	1.11 (0.82 – 1.49)	9.1 (-22.0 – 32.9)

*Abbreviations*: *AR* attributable risk, *CI* confidence interval, *eGFR* Estimated Glomerular Filtration Rate, *HR* hazard ratio

^a^Non-natural causes of death (ICD-10 codes: S00-T98) excluded; ^b^Estimated using Fine-Gray models adjusted for sex, age at enrolment, years of education, HBsAg status, presence of hyperglycaemia (fasting plasma glucose > 126 mg/dL) or reduced kidney function (eGFR< 60 mL/min/1.73m^2^), and FIB-4 index score; ^c^Calculated from the adjusted Fine-Gray models; ^d^Reference category

The factors associated with all-cause, liver-related, and non-liver-related mortality among people positive for anti-HCV are reported in Table 3. Gender was associated with an increased risk of death from non-liver-related causes (HR = 1.55, 95% CI: 1.04–2.29 for males vs. females) and -though of borderline statistical significance- all causes (HR = 1.39, 95% CI: 0.99–1.96), but not with increased liver-related mortality. Similarly, age at enrolment was found to be associated with approximately 2-fold increased risk of death due to all causes and to non-liver-related conditions (HR = 1.86, 95% CI: 1.48–2.35 and HR = 2.09, 95% CI: 1.55–2.83 for any 10-year increase in age, respectively). People infected with HCV with reduced kidney function had a 2.5-fold higher risk of death from non-liver-related causes (95% CI: 1.36–4.62) than those without this disorder. This effect was observed in particular for mortality from circulatory system diseases (HR = 2.35, 95% CI: 1.02–5.41; data not shown). Moreover, although the small sample size calls for caution in interpretation, the presence of both hyperglycaemia and reduced kidney function was significantly associated with liver-related mortality (HR = 5.83, 95% CI: 1.35–25.16). High FIB-4 index score was significantly associated to all-cause (HR = 1.96, 95% CI: 1.00–3.82 for > 3.25 vs. < 1.45) and liver-related mortality (HR = 39.96, 95% CI: 4.73–337.54).

**Table 3:** HRs of death for all, liver-related and non-liver-related causes among positive people to antibodies against HCV (anti-HCV^+^) according to selected variables

Characteristics	All-cause deaths^**b**^ (***n*** = 154)		Liver-related deaths (***n*** = 41)		Non-liver-related deaths^**b**^ (***n*** = 113)	
	N (%)	HR (95% CI)^c^	N (%)	HR (95% CI)^c^	N (%)	HR (95% CI)^c^
Sex						
Female	64 (36.8)	1^d^	19 (10.9)	1^d^	45 (25.9)	1^d^
Male	90 (55.6)	1.39 (0.99–1.96)	22 (13.6)	0.95 (0.47–1.92)	68 (42.0)	1.55 (1.04–2.29)
Age at enrolment						
Increase of 10 years	154 (45.8)	1.86 (1.48–2.35)	41 (12.2)	1.09 (0.77–1.54)	113 (33.6)	2.09 (1.55–2.83)
Hyperglycaemia/reduced kidney function^a^						
None	105 (40.4)	1^d^	32 (12.3)	1^d^	73 (28.1)	1^d^
Hyperglycaemia only	15 (40.5)	0.83 (0.48–1.43)	5 (13.5)	0.79 (0.26–2.40)	10 (27.0)	0.79 (0.40–1.58)
Reduced kidney function	28 (90.3)	1.54 (0.92–2.60)	2 (6.5)	0.32 (0.08–1.29)	26 (83.9)	2.51 (1.36–4.62)
Both	5 (71.4)	1.86 (0.76–4.57)	2 (28.6)	5.83 (1.35–25.16)	3 (42.9)	0.79 (0.22–2.83)
FIB-4 index score^a^						
< 1.45	19 (18.5)	1^d^	1 (1.0)	1^d^	18 (17.5)	1^d^
1.45–3.25	78 (50.3)	1.25 (0.71–2.19)	12 (7.7)	6.66 (0.79–56.51)	66 (42.6)	0.86 (0.46–1.59)
> 3.25	56 (72.7)	1.96 (1.00–3.82)	28 (36.4)	39.96 (4.73–337.54)	28 (36.4)	0.49 (0.21–1.17)

*Abbreviations*: *CI* confidence interval, *eGFR* Estimated Glomerular Filtration Rate, *HR* hazard ratio

^a^The sum does not add up to the total because of missing values; ^b^Non-natural causes of death (ICD-10 codes: S00-T98) excluded; ^c^Estimated using Fine-Gray models adjusted for sex, age at enrolment, presence of hyperglycaemia (fasting plasma glucose > 126 mg/dL) or reduced kidney function (eGFR< 60 mL/min/1.73m^2^), FIB-4 index score, and HCV RNA status; ^d^Reference category

## Discussion

The results from this population-based cohort study indicated that HCV infection was associated with all-cause and liver-related mortality, highlighting the favourable role of prevention and treatment of HCV infection. We found that individuals positive to antibodies against HCV, and in particular those with chronic infection were at higher risk of all-cause and liver-related deaths than those who were anti-HCV negative, suggesting that approximately 34% of all-cause deaths and 85% of liver-related deaths may be attributable to chronic HCV infection. On the contrary, no increased risk emerged among those with cleared the infection.

Several studies have sought to quantify the burden of mortality from HCV [24–31]. Most of them -although remarkably different in design, in the number of patients, and in length of follow-up- revealed elevated mortality associated with HCV infection, as measured by anti-HCV seropositivity alone [25–28, 31]. In line with other studies [29, 32], we observed that only anti-HCV positive individuals with circulating HCV RNA had an increased risk of dying from all causes of death, whereas the risk for anti-HCV seropositive people with negative HCV RNA was similar to that for anti-HCV seronegative ones. These results implied that patients with active viral infection may consistently benefit from antiviral treatment to reduce their overall mortality.

The assessment of mortality among people with HCV should comprise the characterization of cause-specific mortality. It is well recognized that HCV may cause fatal liver diseases, including liver cirrhosis and HCC. Our results are in agreement with several studies that showed a strong association between HCV infection and liver-related mortality [24–27, 29, 30], this association was evident only in individuals with chronic infection who had a significantly higher risk of dying from liver-related diseases than anti-HCV-negative ones. To this regard, our previous study also documented an elevated incidence of HCC in individuals with chronic HCV infection as compared to uninfected ones [15], emphasizing the importance of active infection in predicting also long-term mortality risks from liver conditions. Indeed, it has been shown that patients with cleared HCV infection presented a lower risk of liver fibrosis and, therefore, a presumably lower risk of death [33, 34].

The association between HCV infection and the risk of death from any cause other than liver disease has been frequently reported with conflicting results. Our findings of no increased risk are consistent with those of a large US study using data from the Third National Health and Nutrition Examination Surveys [24]. Similarly, a cohort study from Denmark reported no substantial association between chronic HCV-infection and an increase in non-liver related mortality overall after adjustment for potential confounders [34]. On the other hand, few investigations have shown an increased risk of non-liver-related deaths in patients infected with HCV, probably explained by the presence of comorbidities, alcohol abuse, and injecting drug use in the HCV-infected population [35].

In the present study, most of the deaths from non-liver-related causes were ascribed to circulatory system diseases (238/550, 43%), malignant neoplasms excluding liver cancer (171/550, 31%), respiratory system diseases (42/550, 8%), and diabetes (40/550, 7%) (see Supplementary Table 2), even though they were not significantly associated with HCV (data not shown). In contrast, other investigations have demonstrated significant associations between HCV and mortality from a series of extra-hepatic conditions, including renal and cardiovascular diseases [27, 29, 36]. Previous clinical studies reported an elevated prevalence of metabolic disorders in HCV patients compared to uninfected controls [37]. However, other factors, including high-risk behaviours, lower socioeconomic status, and genetic predisposition, may have also played a role in non-liver-related mortality [24].

A significant burden of comorbidities in patients with HCV and, in particular, elevated risk of death in presence of diabetes, chronic renal diseases, or cardiovascular diseases have been previously found in other studies [17, 38]. Our analysis conducted in the subgroup of people infected with HCV found that non-liver-related mortality -particularly mortality from circulatory system diseases- was increased among individuals with reduced kidney function, while no association emerged in people with hyperglycaemia. Furthermore, the risk of liver-related deaths was elevated in the presence of both disorders, but results should be interpreted with caution since they were based on a low number of events (*n* = 2).

We also observed that high FIB-4 index score was associated with both all-cause and liver-related mortality among anti-HCV-positive people. In line with our results, a recent investigation of HCV patients showed that the risk of liver-related deaths increased significantly with elevated FIB-4 scores, suggesting that the FIB-4 index could be an important tool to evaluate liver disease risk profile and treatment prioritization [39]. Moreover, in a cohort of HIV-infected patients, the FIB-4 was found to be a risk factor for liver-related deaths, regardless of infection with HCV [40].

It is worth noting that a general decline in HCV prevalence in the same area of this study has been reported in recent years [41]. This is mostly due to the disappearance of the first and most consistent wave of HCV epidemics in Italy in the 1950s and 1960s at least partly explained by the excess mortality among older persons with HCV which is consistent with our results. Nevertheless, as we have already published, a non-negligible prevalence of undiagnosed HCV infection may still be present among older persons, especially in low socioeconomic areas, and it can be speculated that among them a sizeable population of persons with advanced fibrosis, who should be rapidly treated with the new direct antiviral agents, may exist in southern Italy [42].

Given that treatment with DAAs induces the elimination of HCV in over 98% of cases, and in view of the WHO program to eradicate the infection by 2030, not treating HCV patients is currently considered unethical and illegal. One way to assess the impact of treatment on HCV disease is to evaluate the events that occur before and after treatment with DAAs and the results herein reported could represent a reliable historical assessment useful for the evaluation of the effect of universal DAA provision. Growing literature is providing new insights on the effect of DAAs, showing that a sustained viral response by DAA is able to reduce the risk of developing diabetes [43], renal function [44], and overall cardiovascular events’ risk [45]. Since the extensive introduction of DAAs in several countries, recent evidence has shown a sharp decline in liver disease morbidity and overall mortality, as in the UK [6] and Australia [7], reversing the increasing trend observed before their introduction. Therefore, similar results could be observed also in Italy in the near future; indeed, from 2015 to March 2021, over 220,000 DAA-treatments have been administered according to the National Registry data from the Italian Medicines Agency (AIFA), giving reasons to forecast that Italy could meet WHO targets by 2030 [2]. A recent study on HCV burden and treatment trends in European countries reported that in Italy the number of cured individuals will exceed the number of patients with infection by 2026 [46]; however, despite the availability and the increasing use of DAAs, still a large number of patients remained undiagnosed and untreated in the analysed countries [46].

Some limitations should be taken into account when interpreting study results. First of all, as for most studies involving data derived from population seroprevalence surveys, we could not rule out the possibility of sampling bias, as people who refused to take part in the study may be systematically different from those who participated, and sampling weights were not applied in the analyses. Nevertheless, the impact of non-participation on prevalence estimates of HCV/HBV infection has been already discussed in our previous paper [14].

The lack of information about alcohol consumption -a known risk factor in the progression of liver disease- prevented the evaluation of its potential confounding effect on cause-specific mortality. It is known that people with HCV infection are more likely to drink alcohol than those without HCV [47]. Thus, excessive alcohol consumption could partly explain the poor prognostic outcomes and liver-related deaths in HCV-infected patients [48–50]. Nevertheless, in southern Italy, HCV infection has been shown to be the major aetiological factor observed in cirrhotic patients over the last decades [13], a finding widely divergent from that observed in northern Italy and in northern Europe, where excessive alcohol consumption accounted for most cases of liver cirrhosis [13, 51].

Another important limitation is the lack of complete information about antiviral treatment. Indeed, such information was available in less than half (*n* = 118) of the 246 HCV-RNA positive subjects (i.e., those who were aware of their serostatus at enrolment). Among them, only 42 (35.6%) had received conventional interferon-based treatment at the time of testing. Although the relatively small proportion of individuals being treated makes it unlikely that our results are substantially biased by the lack of data on antiviral treatment, we could not exclude an effect of treatment on liver-related mortality.

In addition, information on FIB-4, eGFR, and presence or absence of hyperglycaemia were collected at enrolment, making it difficult to ascertain their real contribution to mortality. Likewise, the lack of data on the staging of liver fibrosis -which may have altered during the follow-up period- needs to be borne in mind.

Mortality data relied on the underlying cause of death since other contributing causes are not consistently coded or available to the regional mortality database of people residing in the study area. Although accurate evaluation of mortality outcomes was made possible by the local availability of a high-quality, population-based cancer registry validated by IARC, some misclassification on the specific cause of death cannot be totally excluded.

The limited number of observed events did not guarantee sufficient power to detect associations for specific subgroups and to comprehensively investigate the impact of HCV on specific causes of non-liver related mortality, thus potential associations may have been hidden, calling for caution in the interpretation of the results.

Despite these limitations, our study -which involved a large cohort of 4492 people living in an area with a high prevalence of HCV infection- yielded important information on the effect of HCV on cause-specific mortality before the advent of DAAs. The use of population-based data that allowed an accurate evaluation of mortality outcomes, the length of follow-up, and accurate testing for serological detection of HCV (including HCV RNA testing) represent major strengths of our study.

## Conclusion

In conclusion, this population-based cohort study further supports the detrimental impact of HCV infection on all-cause and, particularly, liver-related mortality. This effect was evident among people with chronic HCV infection, while those with cleared infection had the same risk of uninfected ones. These results provide additional insights into the need to identify through screening all people with chronic HCV infection, notably in areas with a high prevalence of infection with HCV, and to promptly provide them with DAAs treatment in order to progressively achieve WHO target for HCV elimination and further reduction of HCV-related mortality [52].

## Supplementary Information

**Additional file 1: Supplementary Table 1.** Distribution of 4496 study participants according to selected variables.

**Additional file 2: Supplementary Table 2.** Distribution of causes of death among 4483 study subjects.

## Data Availability

The datasets used and/or analysed during the current study are available from the corresponding author on reasonable request.

## Abbreviations

anti-HCV: antibodies against hepatitis C virus
AR: Attributable risk
CI: Confidence interval
DAA: Direct-Acting Antiviral
eGFR: glomerular filtration rate
HBsAg: Hepatitis B virus surface antigen
HBV: Hepatitis B virus
HCC: Hepatocellular carcinoma
HCV: Hepatitis C virus
HR: Hazard ratio
ICD-10: International Classification of Diseases and Related Health Problems, 10th revision
IQR: Interquartile range
PY: Person-year
