# Supplementary material for: Hepatitis C virus infection and risk of liver-related and non-liver-related deaths: a population-based cohort study in Naples, southern Italy
Source: BMC Infect Dis. 2021 Jul 8;21:667. doi: 10.1186/s12879-021-06336-9 (PMC8268172; doi:10.1186/s12879-021-06336-9)
Supplement: Supplementary file 1 — Additional file 1: Supplementary Table 1. Distribution of 4496 study participants according to selected variables. [file 12879_2021_6336_MOESM1_ESM.docx]

**Supplementary Table 1.** Distribution of 4496 study participants according to selected variables

|  | **All** | |  | **Anti-HCV^+^** | |
| --- | --- | --- | --- | --- | --- |
|  | **(n=4496)** | |  | **(n=336)** | |
| **Characteristics** | N | col% |  | N | row% |
|  |  |  |  |  |  |
| Sex |  |  |  |  |  |
| Female | 2482 | 55.2 |  | 174 | 7.0 |
| Male | 2014 | 44.8 |  | 162 | 8.0 |
| Age at enrollment (years) |  |  |  |  |  |
| <50 | 769 | 17.1 |  | 39 | 1.5 |
| 50-59 | 793 | 17.6 |  | 56 | 7.6 |
| 60-69 | 727 | 16.2 |  | 97 | 17.6 |
| ≥70 | 800 | 17.8 |  | 144 | 24.4 |
| Education (years)^a^ |  |  |  |  |  |
| <9 | 2699 | 61.9 |  | 268 | 9.9 |
| ≥9 | 1661 | 38.1 |  | 48 | 2.9 |
| History of HCV testing^a^ |  |  |  |  |  |
| No | 3040 | 77.6 |  | 128 | 4.2 |
| Yes | 877 | 22.4 |  | 172 | 19.6 |
| Living with a person known to be infected with HCV^a^ |  |  |  |  |  |
| No | 3045 | 82.5 |  | 194 | 6.4 |
| Yes | 644 | 17.5 |  | 69 | 10.7 |
| Sexual intercourse with HCV-positive partner^a^ |  |  |  |  |  |
| No | 3657 | 97.4 |  | 238 | 6.5 |
| Yes | 98 | 2.6 |  | 29 | 29.6 |
| Use of intravenous drugs^a^ |  |  |  |  |  |
| No | 3957 | 99.5 |  | 282 | 7.1 |
| Yes | 18 | 0.5 |  | 5 | 27.8 |
| History of blood transfusion^a^ |  |  |  |  |  |
| No | 4226 | 95.4 |  | 275 | 6.5 |
| Yes | 202 | 4.6 |  | 50 | 24.8 |
| History of surgery^a^ |  |  |  |  |  |
| No | 1711 | 38.6 |  | 94 | 5.5 |
| Yes | 2720 | 61.4 |  | 231 | 8.5 |
| History of prolonged dental therapies^a^ |  |  |  |  |  |
| No | 2923 | 66.4 |  | 190 | 6.5 |
| Yes | 1477 | 33.6 |  | 131 | 8.9 |
|  |  |  |  |  |  |

^a^The sum does not add up to the total because of missing values.
