# Supplementary material for: Hepatitis C virus infection and risk of liver-related and non-liver-related deaths: a population-based cohort study in Naples, southern Italy
Source: BMC Infect Dis. 2021 Jul 8;21:667. doi: 10.1186/s12879-021-06336-9 (PMC8268172; doi:10.1186/s12879-021-06336-9)
Supplement: Supplementary file 2 — Additional file 2: Supplementary Table 2. Distribution of causes of death among 4483 study subjects. [file 12879_2021_6336_MOESM2_ESM.docx]

**Supplementary Table 2.** Distribution of causes of death among 4483 study subjects.

| **Causes of death (ICD-10)** | | **Total**  **(4483)** | **anti-HCV^─^**  **(N=4147)** | **anti-HCV^+^**  **(N=336)** | **anti-HCV^+^ and HCV RNA^─^**  **(N=90)** | **anti-HCV^+^ and HCV RNA^+^**  **(N=246)** |
| --- | --- | --- | --- | --- | --- | --- |
|  |  |  |  |  |  |  |
| **All-causes** | **All (A00-T98)** | **626** | **469** | **157** | **32** | **125** |
| **Non-natural causes** | **All (S00-T98)** | **20** | **17** | **3** | **1** | **2** |
| **Natural causes** | **All (A00-R99)** | **606** | **452** | **154** | **31** | **123** |
| **Liver-related** | **All liver-related** | **56** | **15** | **41** | **3** | **38** |
|  | *Liver cancer (C22)* | *24* | *4* | *20* | *1* | *19* |
|  | *Chronic Hepatitis (K73)* | *4* | *2* | *2* | *0* | *2* |
|  | *Cirrhosis (K74)* | *28* | *9* | *19* | *2* | *17* |
| **Non-liver-related** | **All non-liver-related but S00-T98** | **550** | **437** | **113** | **28** | **85** |
|  | All malignant neoplasms (C00-C97 but C22) | 171 | 138 | 33 | 9 | 24 |
|  | *Stomach (C16)* | *6* | *4* | *2* | *1* | *1* |
|  | *Colon-rectum (C18-C20)* | *11* | *10* | *1* | *0* | *1* |
|  | *Pancreas (C25)* | *12* | *12* | *0* | *0* | *0* |
|  | *Lung (C34)* | *46* | *35* | *11* | *3* | *8* |
|  | *Breast (C50)* | *8* | *8* | *0* | *0* | *0* |
|  | *Prostate (C61)* | *14* | *10* | *4* | *1* | *3* |
|  | *Bladder (C67)* | *10* | *7* | *3* | *2* | *1* |
|  | *Central nervous system (C71)* | *7* | *7* | *0* | *0* | *0* |
|  | *Non-Hodgkin Lymphoma (C82-C85, C96)* | *4* | *3* | *1* | *0* | *1* |
|  | *Multiple Myeloma (C90)* | *5* | *4* | *1* | *1* | *0* |
|  | *Leukaemia (C91-C95)* | *11* | *9* | *2* | *0* | *2* |
|  | Diabetes (E10-E14) | 40 | 32 | 8 | 1 | 7 |
|  | Circulatory system diseases (I00-I99) | 238 | 187 | 51 | 10 | 41 |
|  | *Hypertensive diseases (I10-I15)* | *34* | *26* | *8* | *3* | *5* |
|  | *Ischemic heart diseases (I20-I25)* | *89* | *70* | *19* | *4* | *15* |
|  | *Cerebrovascular diseases (I60-I69)* | *54* | *40* | *14* | *2* | *12* |
|  | Respiratory system diseases (J00-J99) | 42 | 34 | 8 | 2 | 6 |
|  |  |  |  |  |  |  |

Abbreviations: ICD-10, International classification of diseases and Related Health Problems 10^th^ revision.
